# Oxygen harvesting from carbon dioxide: simultaneous epoxidation and CO formation†

**DOI:** 10.1039/d1sc04209b

**Published:** 2021-09-13

**Authors:** Han Xu, Muhammad Shaban, Sui Wang, Anas Alkayal, Dingxin Liu, Michael G. Kong, Felix Plasser, Benjamin R. Buckley, Felipe Iza

**Affiliations:** Wolfson School of Mechanical, Electrical and Manufacturing Engineering, Loughborough University Loughborough Leicestershire LE11 3TU UK f.iza@lboro.ac.uk; School of Aerospace Science and Technology, Xidian University Xi'an 710071 China; State Key Laboratory of Electrical Insulation and Power Equipment, Centre for Plasma Biomedicine, Xi'an Jiaotong University Xi'an 710049 China; Department of Chemistry, Loughborough University Loughborough Leicestershire LE11 3TU UK b.r.buckley@lboro.ac.uk; Division of Advanced Nuclear Engineering, Pohang University of Science and Technology (POSTECH) Pohang Gyeongbuk 790-784 South Korea

## Abstract

Due to increasing concentrations in the atmosphere, carbon dioxide has, in recent times, been targeted for utilisation (Carbon Capture Utilisation and Storage, CCUS). In particular, the production of CO from CO_2_ has been an area of intense interest, particularly since the CO can be utilized in Fischer–Tropsch synthesis. Herein we report that CO_2_ can also be used as a source of atomic oxygen that is efficiently harvested and used as a waste-free terminal oxidant for the oxidation of alkenes to epoxides. Simultaneously, the process yields CO. Utilization of the atomic oxygen does not only generate a valuable product, but also prevents the recombination of O and CO, thus increasing the yield of CO for possible application in the synthesis of higher-order hydrocarbons.

## Abstract 2

Selective formation of atomic oxygen to form epoxides in a waste free process is reported. Simultaneously generating carbon monoxide from carbon dioxide for further use.

## Graphical abstract

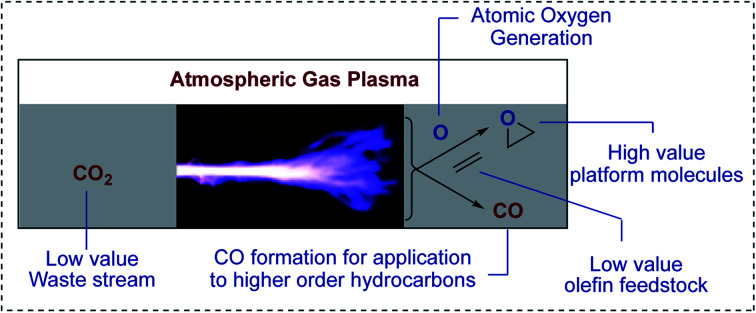

## Introduction

Given the urgent need to reduce greenhouse gas emissions and the growing concerns about the environmental impact of chemical processes, carbon dioxide (CO_2_) utilisation has received growing attention in recent years.^1–5^ The idea of using CO_2_ as a carbon source for organic synthesis is not new and indeed CO_2_ has been used in the manufacture of salicylic acid, urea and cyclic carbonates for 50–100 years. However, due to its relative inertness (Δ_f_*H*° of −394 kJ mol^−1^), these processes are significantly energy demanding, with reactions typically taking place at high temperatures and pressures. Under mild conditions, efficient chemical incorporation of CO_2_ is restricted to reactive substrates, such as epoxides to produce cyclic carbonates^6,7^ and amines to produce carbamates,^8^ often in the presence of catalysts.^9–11^

In fact, artificial photosynthesis, *i.e.* the transformation of CO_2_ and water to chemical fuels, has been pursued for more than three decades.^1,2^ Different electrochemical, photochemical and electrocatalytic processes have been proposed but despite recent advances, these processes remain non-viable from a techno-economical viewpoint. As in natural photosynthesis, reduction of CO_2_ in these processes leads to the release of oxygen and/or water. However, it occurred to us that if it were possible to split carbon dioxide and utilize the oxygen atom (O) to generate a value-added material, one could effectively generate an oxidation process while simultaneously producing carbon monoxide as a high-value energy-rich product. Furthermore, atomic oxygen would be a green oxidant as it would not generate an oxidant waste stream. This is of interest because oxidation processes frequently lead to the generation of large waste streams. For example, the widely used oxidant Oxone™ produces around 25 kg of waste per kg of oxygen transferred.

**Fig. 1:**
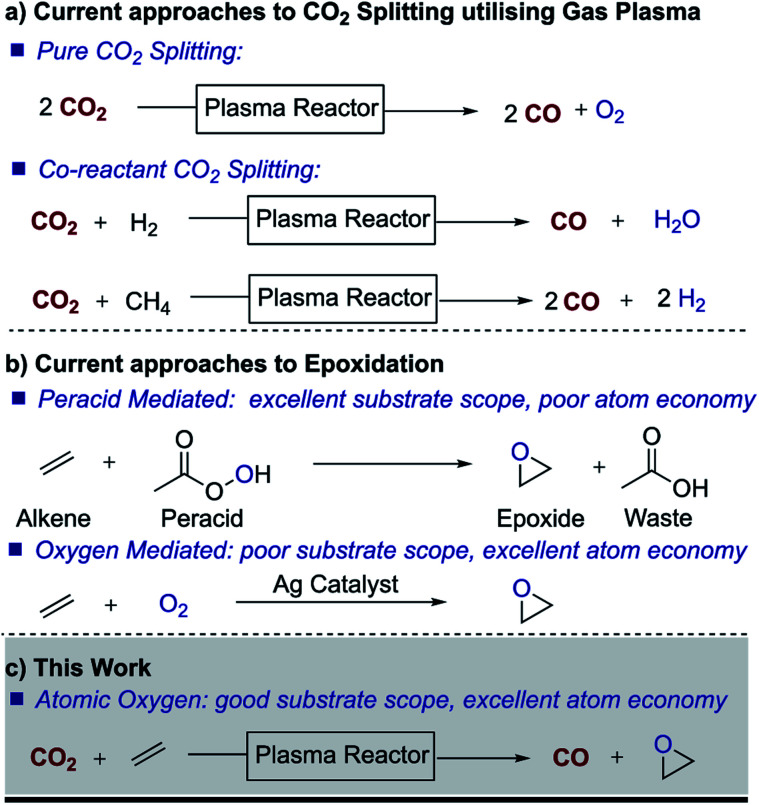
Current routes to plasma CO_2_ utilisation and epoxidation.

Besides electrochemical and photochemical processes, nonthermal gas plasma has emerged in recent years as a technology with great potential for CO_2_ splitting (Fig. 1).^12^ As electrochemical and photochemical processes, plasma processes can be driven by renewable sources. The technology is also scalable and in fact, low-pressure nonthermal plasmas have already been successfully used for the dissociation of molecular oxygen in materials applications for decades. Nonthermal plasmas are a well-established large-scale manufacturing technique widely exploited, for example, for deposition and etching in the semiconductor industry and in ozone generation plants.^13,14^ Recent plasma research on CO_2_ splitting has focussed on optimizing the dissociation process, and therefore, the different mechanisms that lead to the dissociation of CO_2_ into CO and O in a non-thermal plasma are relatively well understood.^15–17^

Traditionally, however, the use of plasma for chemical synthesis has been limited by the incompatibility of the vacuum requirements of conventional plasma systems with the vapor pressure limitations of liquid chemicals, as well as the batch nature of vacuum processing. Nonetheless, in recent years, advances in the generation of non-thermal plasmas at atmospheric pressure have opened the possibility of plasma treatment of liquids and substrates which are not vacuum compatible.^18^ In this work, we demonstrate the use of nonthermal atmospheric pressure gas plasma to split CO_2_ and the possibility of creating a dual process in which CO_2_ is reduced to CO while serving as a source of atomic O for synthesis purposes. Of all the possible oxidation processes, we focused on epoxidation, a large scale and important oxidation process for most chemical industries. Epoxides are key building blocks and important intermediates in the preparation of many products, including drugs, paints, adhesives, sealants, plastics, *etc.* Owing to the lack of reactivity of small molecular oxygen donors and alkenes, peroxy acids such as *meta*-chloroperoxybenzoic acid (*m*CPBA) are often used to drive epoxidation reactions, which leads to processes with poor atom economy and halogenated waste streams. We therefore embarked on initial studies to trap atomic oxygen with an alkene in order to generate an epoxide, thereby targeting the important industrial area of oxidation.

## Results and discussion

Carbon dioxide was dissociated into CO and O in a radio frequency plasma COST jet (ESI Fig. S1†), a plasma jet designed as a reference source for research of non-equilibrium atmospheric pressure plasmas as part of a European Cooperation in Science and Technology (COST) initiative.^19^ This jet has also been shown to be amenable for the study of plasma–liquid interactions at atmospheric pressure^20,21^ and provides a well-characterized device that allows the comparison of results among laboratories in different countries. Previous studies have demonstrated the ability of this device to produce atomic oxygen from admixtures of molecular oxygen. Two-photon absorption laser-induced fluorescence (TALIF) and mass spectrometry (MS) experiments have measured typical O concentrations in excess of 10^14^ cm^−3^ in the plasma afterglow at 1–2 cm from the device nozzle.^22,23^ A recent study with oxygen isotopes has also demonstrated the possibility of transferring atomic O generated in the plasma into a solution, where it can react with organic substrates.^20^

In this study, *trans*-stilbene and *cis*-stilbene solutions were (independently) exposed to the afterglow of the plasma and Fig. 1 shows the gas chromatographs of both solutions after 60 min plasma exposure. The chromatographs reveal that indeed it is possible to epoxidise stilbene with oxygen that originates from CO_2_. Naphthalene was added to the solutions post-treatment to provide a reference for quantitative analysis.^24^ In both cases, the same products were identified: the desired *trans*- and *cis*-epoxides and two additional carbonyl compounds, 2-phenylacetophenone and diphenylacetaldehyde.

**Scheme 1:**
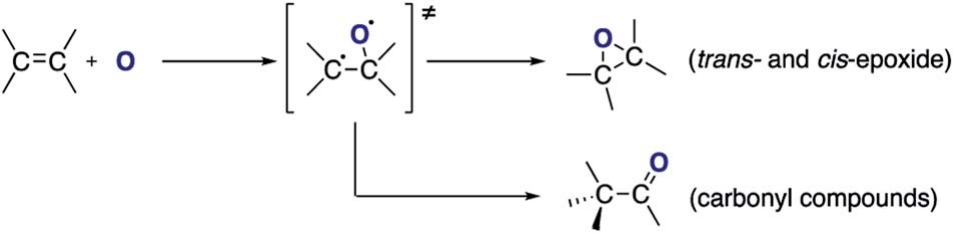
Oxidation of the olefinic double bond by atomic oxygen.

**Fig. 2:**
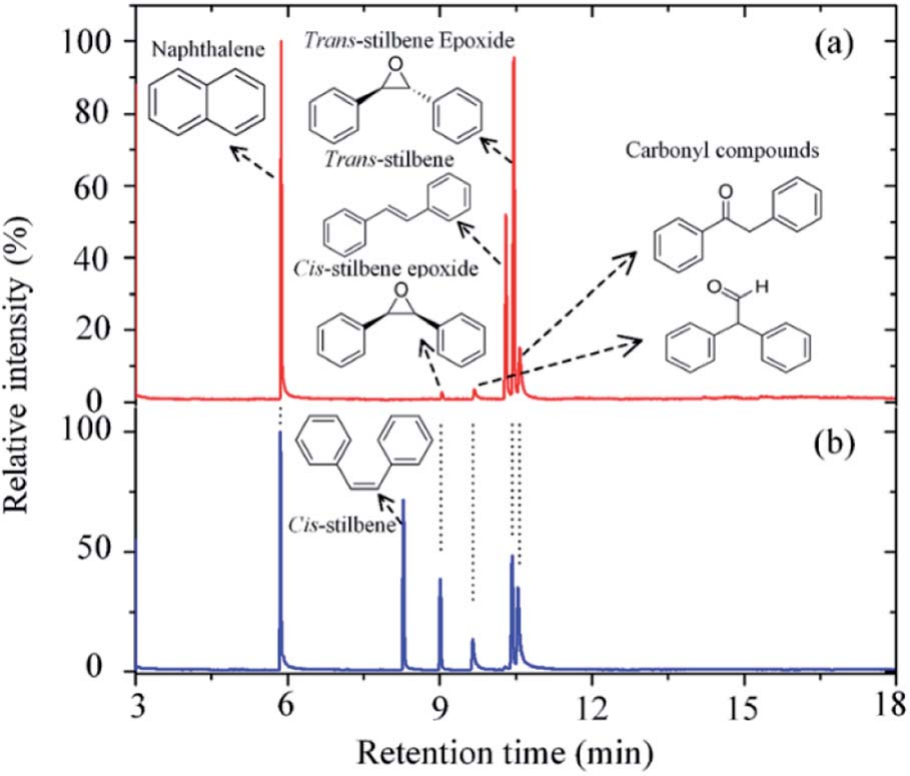
GCMS chromatograph of the *trans*-stilbene solution (a) and *cis*-stilbene solution (b) after exposure to a He + CO_2_ plasma for 60 min at 280 V_rms_, −25 °C and CO_2_ flow rate of 10 sccm.

The formation of *cis*-stilbene epoxide and *trans*-stilbene epoxide in both solutions indicate that the epoxidation process is not stereospecific. Treatment of *trans*-stilbene solutions leads to a small amount of *cis*-stilbene epoxide, which indicates that rotation around the C–C axis of the olefinic bond can occur. No rotation is observed when the solutions are exposed to a pure helium plasma (ESI Fig. S3†), which suggests that the rotation only takes place after atomic oxygen is added to the double bond and an intermediate adduct is formed (Scheme 1). Given the more stable nature of the *trans*-isomer, a larger proportion of *cis*-stilbene is converted to *trans*-stilbene epoxide after exposure to the plasma (Fig. 2).

Quantitative analysis of the chromatographs shown in Fig. 2 indicates that in both cases >55% of the initial stilbene had been oxidized after 60 min of plasma exposure. For the *trans*-stilbene solution, ∼75% of the converted material was the desired epoxide, with the remaining ∼25% being converted to either 2-phenylacetophenone or diphenylacetaldehyde. This is a high yield if one considers that it was obtained under mild conditions without catalyst and without generating an oxidant waste stream. For the *cis*-stilbene solution, the yield was slightly lower at ∼55%, with ∼45% of the *cis*-stilbene being converted to the two carbonyl compounds. The epoxidation process can be applied to other olefin substrates and we have preliminary data for successful epoxidation of a range of alkene classes (aryl, aliphatic α,β-unsaturated); styrene, *trans*-chalcone, β-pinene, and *cis*-cyclooctene (ESI Fig. S5–S7†). In all cases, the main product is the valuable epoxide and the corresponding chromatographs can be found in the ESI.†

Based on the products identified in the chromatographs (Fig. 2), it can be concluded that exposure of the alkene to the split CO_2_ leads to the formation of two main compounds: epoxide and carbonyl compounds (Scheme 1).

**Fig. 3:**
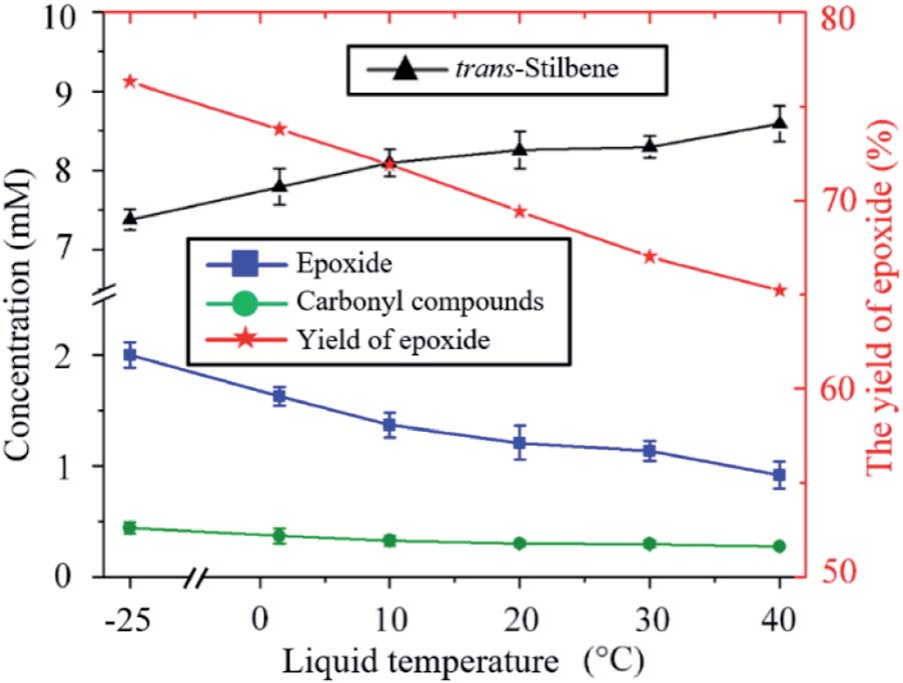
Influence of the temperature on the kinetics of the *trans*-stilbene oxidation after 20 min plasma exposure at 280 V_rms_ and CO_2_ flow rate of 10 sccm.

The ratio of epoxide to carbonyl compounds depends on the conditions in which O is incorporated into the alkene. As shown in Fig. 3, operating at lower temperature favours the formation of epoxide over carbonyl compounds, with the epoxide yield increasing from 65% to 75% as the temperature is decreased from 40 to −25 °C. This is probably a consequence of the more effective quenching of the intermediate adduct at lower temperature.

An indirect influence of the temperature on the kinetics of the process in the current experimental setup is *via* solvent evaporation. As the temperature of the liquid increases, the amount of solvent evaporated during the experiment also increases (ESI Fig. S4†). As a result, the distance between the nozzle of the plasma jet and the surface of the liquid varies over time and increases more significantly at higher temperatures. For example, when the liquid is kept at 40 °C, the distance between the nozzle of the plasma device and the surface of the liquid increases from 4 mm to 25 mm after 20 min. Increasing the distance between the plasma nozzle and the liquid surface causes an increased loss of O atoms as these are then allowed to recombine to either O_2_ or to CO_2_ before reaching the liquid. This causes the decrease in total *trans*-stilbene oxidation shown in Fig. 3 as the temperature increases.

Previous studies of plasma oxidation of olefins employed oxygen as an oxygen donor.^20,21,25^ These studies showed that singlet oxygen did not contribute to the epoxidation of alkenes and that the amount of oxygen that could be put in the system was constrained. In particular, atomic oxygen in the presence of molecular oxygen forms ozone (O_3_), leading to the ozonolysis of the alkene and the preferential formation of aldehyde over the desired epoxide.^21^ As a result, high epoxide yields could only be obtained at low pressure or with small oxygen admixtures (<0.1%). In the present study, this problem is overcome as atomic oxygen is not exposed to molecular oxygen, thereby avoiding the formation of ozone. This is further supported by the lack of benzaldehyde (retention time ∼4 min) in the chromatograms (see Fig. 2).

**Fig. 4:**
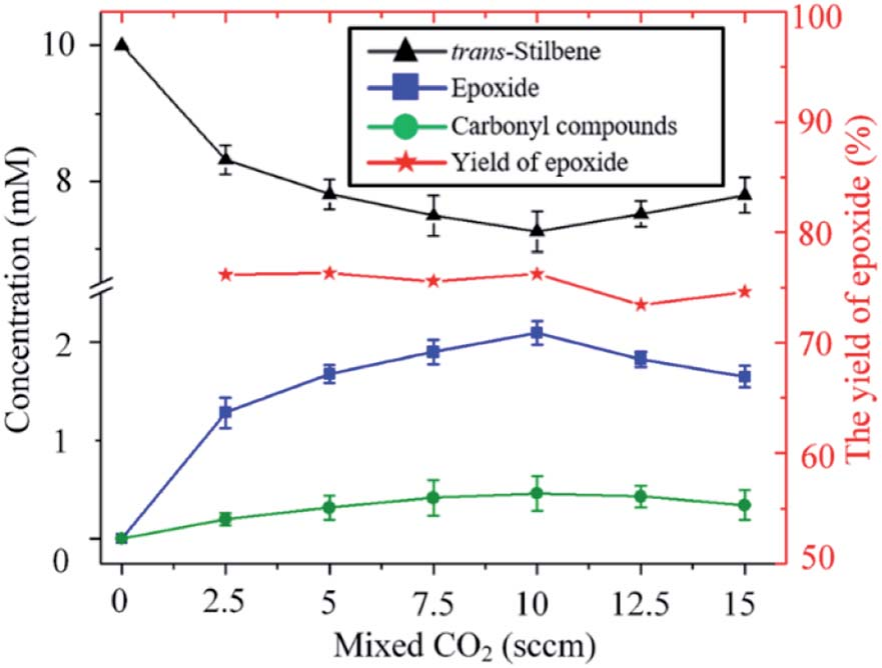
Influence of the carbon dioxide concentration on the kinetics of the *trans*-stilbene oxidation after 20 min plasma exposure at 280 V_rms_ and liquid temperature of −25 °C.

As shown in Fig. 4, for a fixed input voltage, the amount of epoxide produced initially increases with the concentration of CO_2_ in the gas. This is expected as a higher concentration of CO_2_ results in higher production of O. As the CO_2_ continues to increase, however, the plasma density starts to decrease and hence so does the epoxide formation. Operation at higher input power would maintain the plasma density but a different device from the one used in this study would be needed. Importantly, however, the percentage yield of epoxide is insensitive to the concentration of CO_2_ in the feed gas, a distinct and clear advantage of using CO_2_ over O_2_ as an oxygen donor.

**Fig. 5:**
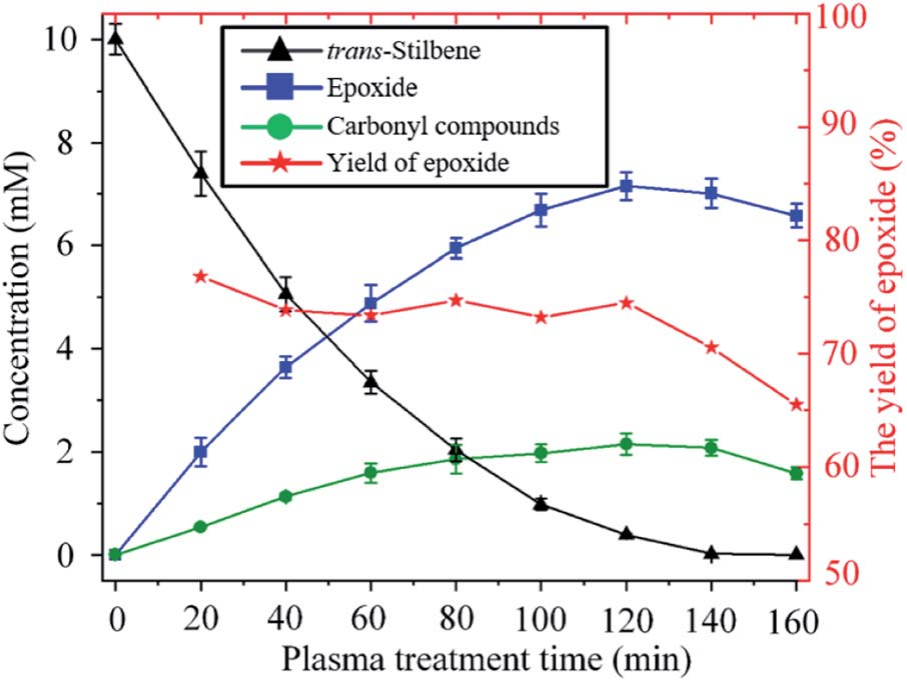
Time evolution of the concentration of *trans*-stilbene, epoxide and carbonyl compounds during exposure to He + CO_2_ plasma (280 V_rms_, −25 °C, 10 sccm CO_2_).

The time evolution of the concentration of *trans*-stilbene, epoxide and carbonyl compounds during the exposure to plasma is shown in Fig. 5. The rate of production of epoxide is largest at the beginning as the concentration of both *trans*-stilbene and atomic oxygen are largest at that time. As *trans*-stilbene is consumed, and the delivery of atomic oxygen decreases due to the evaporation of solvent, the production rate of epoxide decreases over time. Once *trans*-stilbene is consumed, the concentration of epoxide is found to decrease, which implies that atomic oxygen degrades the epoxide. However, since the epoxide yield remains constant at ∼75% while *trans*-stilbene is present, degradation of epoxide can be avoided by operating in excess of *trans*-stilbene.

**Fig. 6:**
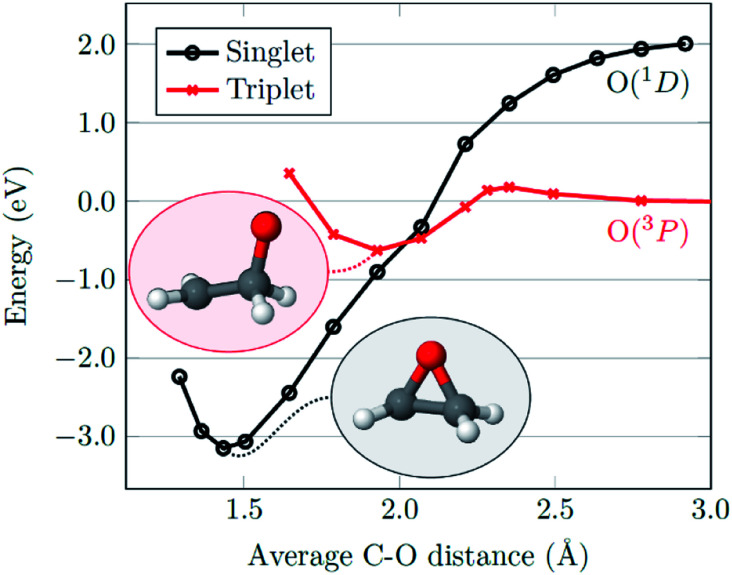
Potential energy curves for an oxygen atom approaching ethene considering singlet (black) and triplet (red) spin computed at the *ab initio* MR-AQCC level of theory by fixing the average C–O distance and relaxing the remaining structure. Molecular structures at the minima of the singlet and triplet surfaces are shown as insets.

**Fig. 7:**
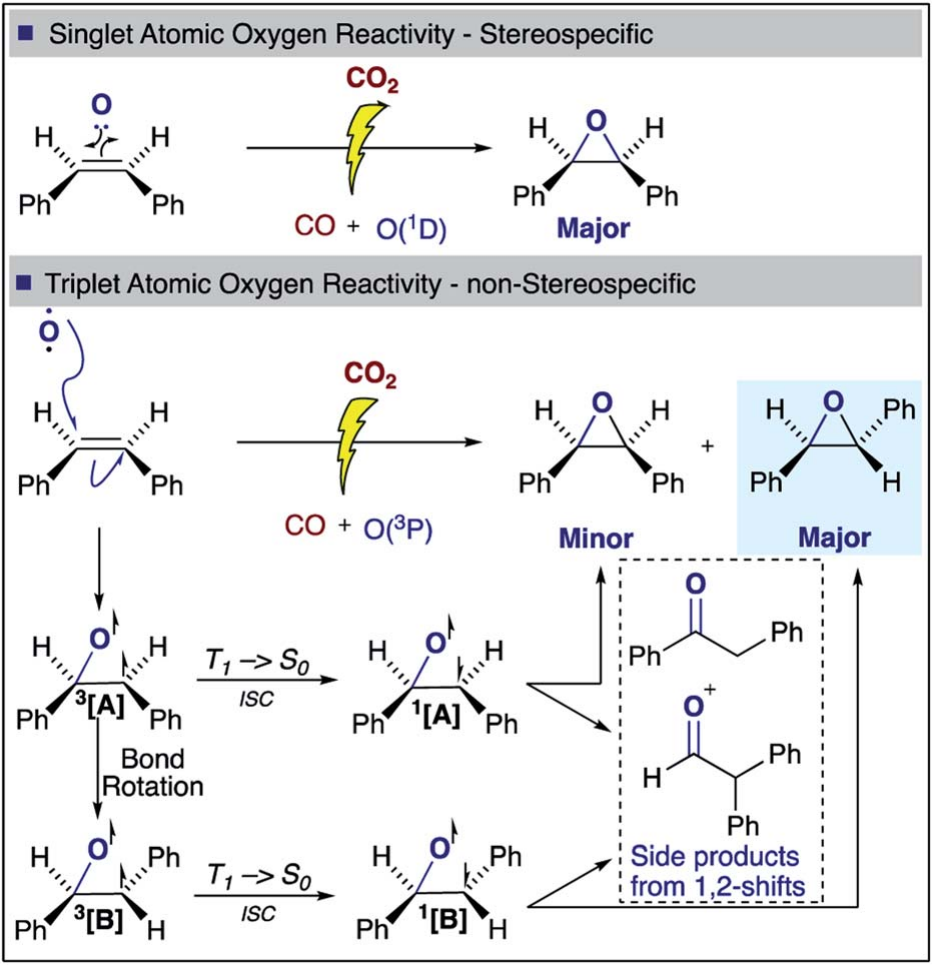
*cis*-Stilbene epoxidation: proposed singlet and triplet mechanisms for the formation of *cis*- and *trans*-stilbene oxide.

With regards to the mechanism of epoxidation, it is clear from the *cis*-stilbene experiment that oxygen transfer is occurring through a non-concerted pathway, as both *cis*- and *trans*-stilbene oxides are produced. *Ab initio* molecular modelling using correlated multireference methods of the active atomic oxygen oxidant approaching ethene reveals the pathways for the epoxide formation (Fig. 6, see ESI† for computational details).^26,27^ Singlet atomic oxygen readily produces epoxide whereas triplet atomic oxygen requires to overcome an energy barrier of ∼0.18 eV. This is a relatively small barrier and many particles in the plasma can provide this energy. We expect the latter to be the main reaction mechanism in our system as the concentration of triplet atomic oxygen (ground state) is higher than that of singlet oxygen in the plasma afterglow. Furthermore, singlet atomic oxygen would readily be quenched by the solvent (see ESI†). In Fig. 6, ground state atomic oxygen approaches from the right on the red triplet line and after overcoming the small energy barrier it binds to ethene to form a biradical intermediate structure (circled in red). This structure enables rotation along the carbon–carbon bond, supporting Scheme 1 and explaining the *cis*/*trans*-isomerisation observed in experiments. In Fig. 7, the biradical intermediate in the case of *cis*-stilbene epoxidation is shown as **3[A]**, and upon carbon–carbon bond rotation it gives **3[B]**. The system then needs to cross over to the singlet (black line, Fig. 6), to produce the epoxide (circled in black). This process could either occur through a monomolecular formally spin-forbidden intersystem crossing process or through bimolecular quenching. In Fig. 7 this is depicted through intersystem crossing (ISC) to form 1,3-singlet diradical **1[B]**, through which subsequent O–C bond formation yields the *anti*-epoxide.^28^

**Fig. 8:**
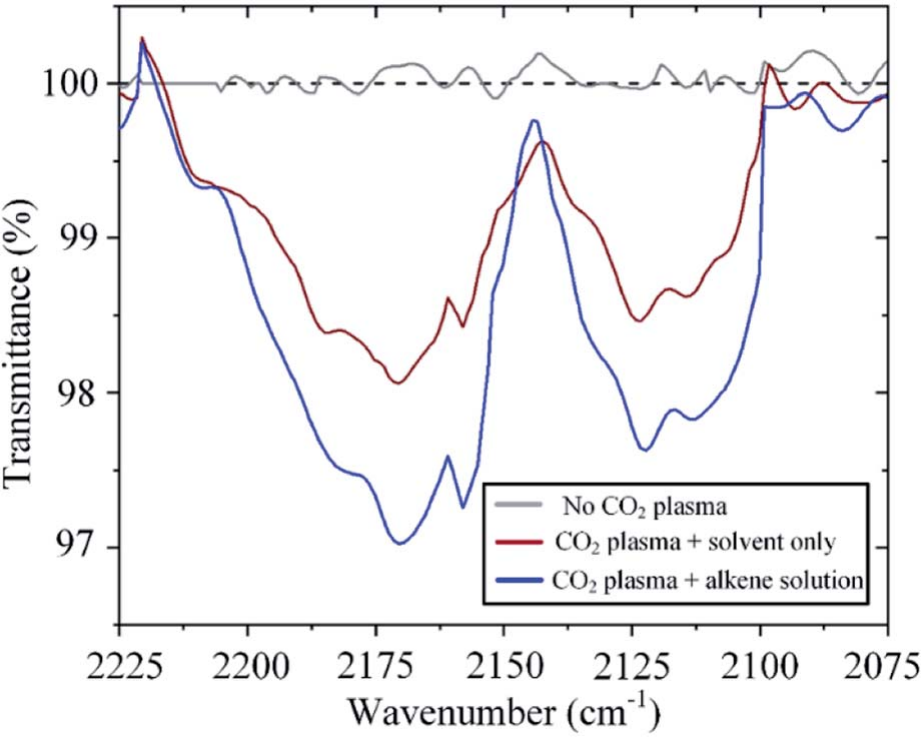
FTIR absorption spectra revealing higher CO concentration in the effluent gas when the gas is exposed to the alkene solution. Absorption at ∼2116 cm^−1^ and ∼2173 cm^−1^ correspond to the ‘P’ branch (Δ*J* = −1, where *J* is the rotational quantum number) and ‘R’ branch (Δ*J* = +1), respectively.

The incorporation of atomic oxygen to the alkene implies that carbon monoxide is also generated in the process. Indeed, Fourier Transform Infrared (FTIR) spectroscopy reveals the presence of CO at the gas outlet of the system. CO is in itself an important feedstock in the synthesis of many chemicals and fuels. For example, hydrogenation of CO can generate methanol and higher-order hydrocarbons through the Fischer–Tropsch process. As expected, the amount of CO detected varies with raising CO_2_ concentration in a similar fashion to that observed for the formation of epoxide (ESI Fig. S2†). Of interest here, however, is the effect of sequestering atomic oxygen from the CO_2_ splitting process to oxidize an alkene. Conventionally, in CO_2_ plasma splitting processes, atomic oxygen is left to recombine, leading not only to the formation of molecular oxygen but also to the back reaction with CO to form CO_2_, thereby having a detrimental effect on the process efficacy.^29^ However, as shown in Fig. 8, the back reaction with CO can be mitigated by sequestering atomic oxygen in a reaction with an alkene, thereby leading to an increase in the CO concentration in the effluent gas under otherwise identical operating conditions. In one particular experiment, the CO concentration increases by 70% when the plasma effluent is exposed to *trans*-stilbene in solution (Fig. 8).

## Conclusions

This study demonstrates the potential use of waste carbon dioxide not only as a source of CO for the synthesis of fuels but simultaneously as a source of atomic oxygen for valuable oxidation reactions. In particular, carbon dioxide was split in a nonequilibrium plasma and the resulting atomic oxygen utilized to oxidize a series of alkenes. The process yields primarily epoxide under mild temperature and pressure conditions without the use of catalysts or co-reagents. The concentration and yield of epoxide increase with decreasing liquid temperature and the efficacy of the process is insensitive to variations of the CO_2_ concentration in the feed gas. Oxidation of *trans*-stilbene produces epoxide with a yield of ∼75% and utilisation of the atomic oxygen in this oxidation process prevents its back reaction with CO, thereby increasing the concentration of CO in the plasma afterglow by ∼70%.

Although scalability of the technology would require recirculation of gases, increased gas–liquid interfacial area and adaptation to flow, the current experimental setup has demonstrated the feasibility for a CO_2_ based green oxidation process with minimal environmental impact and great atom economy.

## Data availability

Data associated with this article, including experimental, computational procedures and compound identification, are available in the ESI.†

## Author contributions

B. R. B., F. I. and H. X. conceived the project; H. X., M. S., S. W. and A. A. conducted the experiments; and the whole team analysed the results. F. P. conducted the computational aspects of the project. B. R. B, F. I., H. X. and F. P. wrote the manuscript with input from the rest of the research team.

## Conflicts of interest

There are no conflicts to declare.

## Supplementary Material

SC-012-D1SC04209B-s001

## References

[cit1] Zhang W., Hu Y., Ma L., Zhu G., Wang Y., Xue X., Chen R., Yang S., Jin Z. (2018). Adv. Sci..

[cit2] Wu J., Huang Y., Ye W., Li Y. (2017). Adv. Sci..

[cit3] Liu Q., Wu L., Jackstell R., Beller M. (2015). Nat. Commun..

[cit4] Olah G. A., Goeppert A., Prakash G. K. S. (2009). J. Org. Chem..

[cit5] Kim Y., Do Park G., Balamurugan M., Seo J., Min B. K., Nam K. T. (2019). Adv. Sci..

[cit6] Clegg W., Harrington R. W., North M., Pasquale R. (2010). Chem.–Eur. J..

[cit7] Martín C., Fiorani G., Kleij A. W. (2015). ACS Catal..

[cit8] Ion A., Parvulescu V., Jacobs P., De Vos D. (2007). Green Chem..

[cit9] Whiteoak C. J., Kielland N., Laserna V., Escudero-Adán E. C., Martin E., Kleij A. W. (2013). J. Am. Chem. Soc..

[cit10] Wu L., Liu Q., Fleischer I., Jackstell R., Beller M. (2014). Nat. Commun..

[cit11] Lu X., Wu Y., Yuan X., Huang L., Wu Z., Xuan J., Wang Y., Wang H. (2018). ACS Energy Lett..

[cit12] Snoeckx R., Bogaerts A. (2017). Chem. Soc. Rev..

[cit13] Oehrlein G. S., Hamaguchi S. (2018). Plasma Sources Sci. Technol..

[cit14] Kogelschatz U. (2003). Plasma Chem. Plasma Process..

[cit15] Urbanietz T., Böke M., Schulz-von der Gathen V., von Keudell A. (2018). J. Phys. D: Appl. Phys..

[cit16] Aerts R., Somers W., Bogaerts A. (2015). ChemSusChem.

[cit17] van Rooij G. J., Den Harder N., Minea T., Berden G., Engeln R., Graswinckel M. F., Zoethout E. (2015). Faraday Discuss..

[cit18] Bruggeman P. J., Kushner M. J., Locke B. R., Gardeniers J. G. E., Graham W. G., Graves D. B., Hofman-Caris R. C. H. M., Maric D., Reid J. P., Ceriani E., Fernandez Rivas D., Foster J. E., Garrick S. C., Gorbanev Y., Hamaguchi S., Iza F., Jablonowski H., Klimova E., Kolb J., Krcma F., Lukes P., Machala Z., Marinov I., Mariotti D., Mededovic Thagard S., Minakata D., Neyts E. C., Pawlat J., Petrovic Z. L., Pflieger R., Reuter S., Schram D. C., Schröter S., Shiraiwa M., Tarabová B., Tsai P. A., Verlet J. R. R., von Woedtke T., Wilson K. R., Yasui K., Zvereva G. (2016). Plasma Sources Sci. Technol..

[cit19] Golda J., Held J., Redeker B., Konkowski M., Beijer P., Sobota A., Kroesen G., Braithwaite N. S. J., Reuter S., Turner M. M., Gans T., O'Connell D., Schulz-von der Gathen V. (2016). J. Phys. D: Appl. Phys..

[cit20] Benedikt J., Mokhtar Hefny M., Shaw A., Buckley B. R., Iza F., Schäkermann S., Bandow J. E. (2018). Phys. Chem. Chem. Phys..

[cit21] Xu H., Wang S., Shaban M., Montazersadgh F., Alkayal A., Liu D., Kong M. G., Buckley B. R., Iza F. (2019). Plasma Processes Polym..

[cit22] Knake N., der Gathen V. S. (2010). Eur. Phys. J. D.

[cit23] Ellerweg D., Benedikt J., von Keudell A., Knake N., Schulz-von der Gathen V. (2010). New J. Phys..

[cit24] GC yields were deterimend from the calibration curve of the desired product, see ESI† for further details and they are based on the amount of starting stilbene

[cit25] Patiño P., Sánchez N., Suhr H., Hernández N. (1999). Plasma Chem. Plasma Process..

[cit26] Szalay P. G., Bartlett R. J. (1993). Chem. Phys. Lett..

[cit27] Lischka H., Shepard R., Müller T., Szalay P. G., Pitzer R. M., Aquino A. J. A., Araújo Do Nascimento M. M., Barbatti M., Belcher L. T., Blaudeau J. P., Borges I., Brozell S. R., Carter E. A., Das A., Gidofalvi G., González L., Hase W. L., Kedziora G., Kertesz M., Kossoski F., Machado F. B. C., Matsika S., Do Monte S. A., Nachtigallová D., Nieman R., Oppel M., Parish C. A., Plasser F., Spada R. F. K., Stahlberg E. A., Ventura E., Yarkony D. R., Zhang Z. (2020). J. Chem. Phys..

[cit28] Strictly speaking, at this point we do not know whether the ISC or the 1,2-shift comes first in the formation of the keto side products

[cit29] Heijkers S., Martini L. M., Dilecce G., Tosi P., Bogaerts A. (2019). J. Phys. Chem. C.

