# Supplementary material for: Oxygen harvesting from carbon dioxide: simultaneous epoxidation and CO formation
Source: Chem Sci. 2021 Sep 13;12(40):13373–8. doi: 10.1039/d1sc04209b (PMC8528036; doi:10.1039/d1sc04209b)
Supplement: SC-012-D1SC04209B-s001 [file SC-012-D1SC04209B-s001.pdf]

Electronic Supplementary Material (ESI) for Chemical Science.
This journal is © The Royal Society of Chemistry 2021

Supporting Information

Oxygen Harvesting from Carbon Dioxide: Simultaneous Epoxidation and CO Formation

Han Xu,[a,b] Muhammad Shaban,[a] Sui Wang,[a,c] Anas Alkayal,[d] Dingxin Liu,[c] Michael G. Kong,[c] Felix Plasser,[d] Benjamin R. Buckley[d]* and Felipe Iza[ae]*

[a] *Wolfson School of Mechanical, Electrical and Manufacturing Engineering, Loughborough University, Loughborough, Leicestershire LE11 3TU, United Kingdom;*
Email: f.iza@lboro.ac.uk
[b] *School of Aerospace Science and Technology, Xidian University, Xi'an 710071, China*
[c] *State Key Laboratory of Electrical Insulation and Power Equipment, Centre for Plasma Biomedicine, Xi'an Jiaotong University, Xi'an 710049, P. R. China;*
[d] *Department of Chemistry, Loughborough University, Loughborough, Leicestershire LE11 3TU, United Kingdom*
Email: b.r.buckley@lboro.ac.uk
[e] Division of Advanced Nuclear Engineering, Pohang University of Science and Technology (POSTECH), Pohang, Gyeongbuk 790-784, South Korea

Table of Contents

A Experimental Setup and Materials 4

A.1. Experimental setup 4

Figure S1. (a) Schematic of the experimental setup. (b) COST plasma jet and (c) custom-made flask used in the experiments. 4

A.2. Materials 5

A.3. Analytical methods 5

A.4. Experimental details 5

A.5. Computational details 7

B Supplementary Figures 8

Figure S2. FTIR spectra of the gases at the exhaust for various experimental conditions. (a) Shows the formation of CO in the plasma in the absence of a liquid solution. (b) Shows that after 30 min the composition of the exhaust gas has reached steady state. (c) Shows that when liquids are introduced in the system, the IR spectra of the exhaust gases is dominated by solvent molecules. Crucially, however, no solvent peaks overlap with the CO absorption bands (2100–2250 cm^{-1}). 9

Figure S3. Chromatographs of *trans*-stilbene and *cis*-stilbene solution after 60 min exposure to a pure helium plasma. Voltage 250 Vrms, solution temperature –25°C. 9

Figure S4. Solvent evaporation during a 20 min plasma treatment as a function of the liquid temperature. Plasma sustained at a voltage of 280 Vrms and with a CO_2 flow rate of 10 sccm. 9

Figure S5. Chromatograph of the *cis*-cyclooctene solution after a 60 min exposure to He+CO_2 plasma. (280 Vrms, –25°C and 10 sccm CO_2) 10

Figure S6. Time evolution of the concentration of *cis*-cyclooctene, cyclooctene oxide and the yield of cyclooctene oxide during exposure to He+CO_2 plasma (280 Vrms, –25°C and 10 sccm CO_2). 10

Figure S7. Chromatographs of (a) styrene, (b) β-pinene and (c) *trans*-chalcone solutions after a 60 min exposure to He+CO_2 plasma. (280 Vrms, –25°C and 10 sccm CO_2) 11

Figure S8. Potential energy curves for an oxygen atom approaching acetonitrile considering singlet (black) and triplet (red) spin computed at the ab initio MR-AQCC level of theory by fixing the average C-O distance and relaxing the remaining structure. The minima on the singlet and triplet surfaces are shown as insets. O1D readily reacts with acetonitrile, thereby quenching O(^{1}D). On the other hand, there is a 1.2eV barrier for O(^{3}P) to react with acetonitrile. This barrier prevents the spontaneous quenching of triplet oxygen by the solvent, making it available for epoxidation of the stilbene. These simulations results, together with the fact that we do not observe cycloaddition of nitrile oxides to the alkene support the idea that O(^{3}P) reacts directly with the alkene and not with the solvent. 12

Figure S9. Retention time and intensity calibration curves for (a) *trans*-stilbene, (b) *trans*-stilbene epoxide, (c) 2-Phenylacetophenone, (d) Diphenylacetaldehyde, (e) *cis*-stilbene, (f) *cis*-stilbene epoxide, (g) *cis*-cyclooctene and (h) cyclooctene oxide. All data obtained from solutions prepared with purified commercial samples (500 µL) and naphthalene (50 mM, 50 µL) as a reference. 15

C References 16

A Experimental Setup and Materials

A.1. Experimental setup

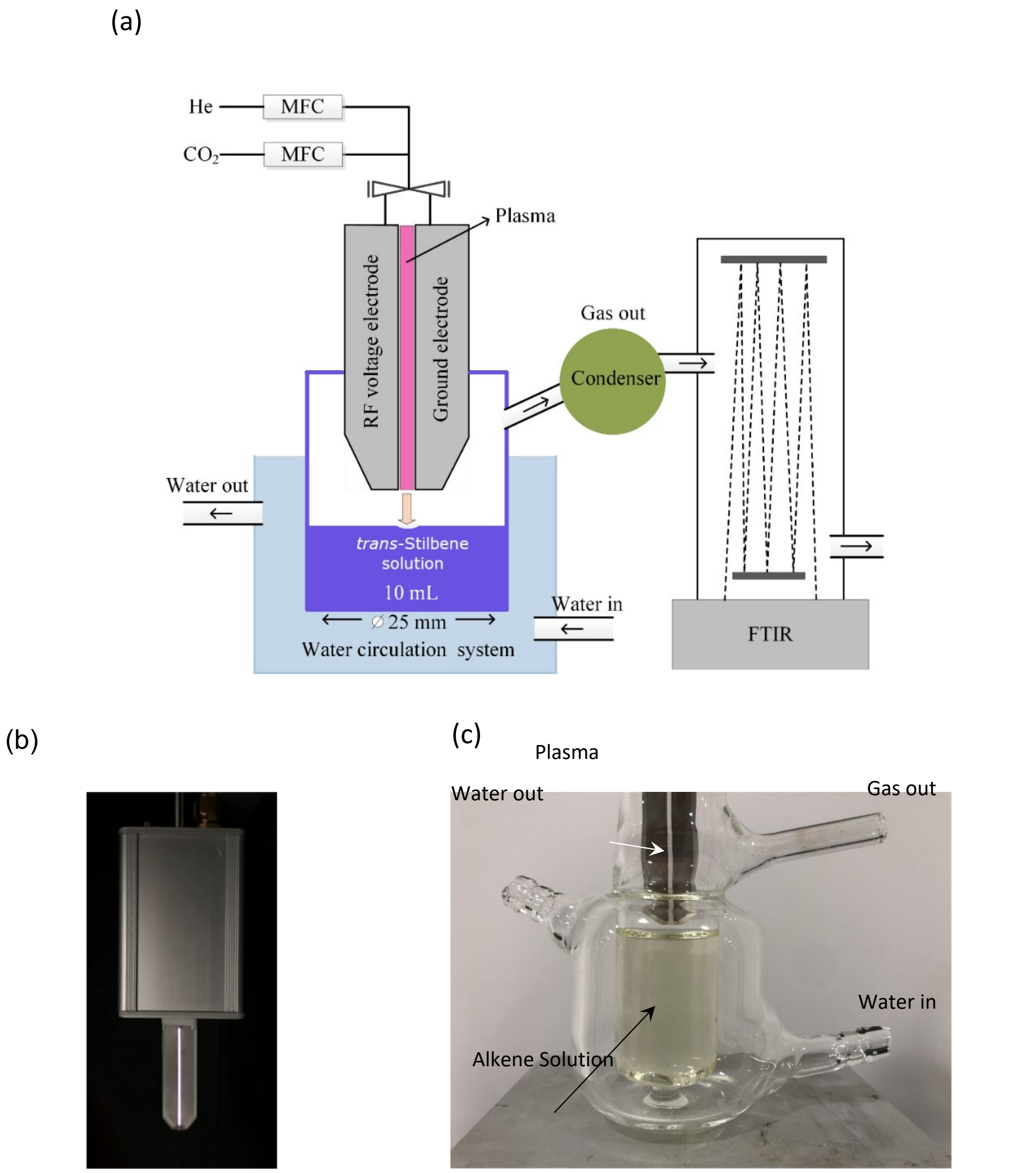

Figure S1. (a) Schematic of the experimental setup. (b) COST plasma jet and (c) custom-made flask used in the experiments.

A.2. Materials

Naphthalene (99%, Sigma Aldrich), *trans*-Stilbene (98%, Alfa Aesar), *trans*-Stilbene oxide (98%, Sigma Aldrich), *cis*-Stilbene (96%, Sigma Aldrich), *cis*-stilbene oxide (97%, Sigma Aldrich), 2-Phenylacetophenone (97%, Sigma Aldrich), Diphenylacetaldehyde (97%, Sigma Aldrich), Acetonitrile (anhydrous, ≥99.8%, VWR Chemicals), Chloroform D (99.8% D, Eurisotop), Ethanol (Anhydrous, KOPTEC USP), *cis*-cyclooctene (cyclooctene, 95%, Sigma Aldrich), Cyclooctene oxide (99%, Sigma Aldrich), (-)-β-Pinene (≥99%, Sigma Aldrich), Styrene (≥99%, Sigma Aldrich), *trans*-chalcone (97%, Sigma Aldrich) were used in the experiments.

A.3. Analytical methods

All infrared spectra were obtained using a Perkin-Elmer Spectrum 65 FT-IR spectrophotometer; thin film spectra were acquired using sodium chloride plates. All ^{1}H and ^{13}C NMR spectra were measured at 400 and 100 MHz using a Bruker Avance 400 MHz spectrometer, a Jeol ECS 400 MHz spectrometer or at 500 and 125 MHz on a Jeol ECZ 500 MHz spectrometer. The solvent used for NMR spectroscopy was $CDCl_3$ (unless stated otherwise) using TMS (tetramethylsilane) as the internal reference.

Analysis by GC-MS utilised a Shimadzu QP2020, GC-2010 Plus, using a 15 m x 0.25 mm DB-5 column and an electron impact low resolution mass spectrometer, Reactions were monitored by GC-MS.

A.4. Experimental details

CO_2 splitting: A capacitively coupled radio-frequency (13.56 MHz) plasma COST jet consisting of two stainless steel electrodes that form a 30 mm long coplanar discharge channel was used to split CO_2. The gas flow rate was 1.4 standard litres per minute (slm) and consisted of an admixture of helium and CO_2 with concentration of up to 1%. A typical *α*-glow mode discharge is formed as a sinusoidal driving voltage of 150–300 V_{rms} is applied to the electrodes, and the gas temperature remains about ten degrees above room temperature. This

is a reference plasma source that has been studied by a number of groups and more information of the device, the discharge characteristics, and its applications can be found elsewhere.[1–5]

Plasma exposure of alkene solutions: Alkenes (*trans*-stilbene, *cis*-stilbene, styrene, *β*-pinene, *trans*-chalcone and *cis*-cyclooctene) were dissolved in acetonitrile to create a 10mM alkene solution. 10 mL samples were then exposed to the plasma. As in previous studies of COST jets treating liquids and to maintain a high flux of O without significantly perturbing the liquid surface, the gap between the jet nozzle and the liquid surface was initially set to 4mm[6,7]. The solution was kept in a flask which had a cooling jacket to control the temperature of the solution during the experiment (Supplementary Figure S1). Water and mixtures of dry ice and ethanol were used in the cooling circuit to vary the temperature of the solution from –25 to +40 ºC. Prior to plasma treatment, the cooling system was run for 10 minutes to equilibrate the temperature of the solution.

Fourier Transform Infrared (FTIR) analysis: The reactor was sealed with the gas exhaust connected to a gas cell (1-16m, Pike technologies) mounted in a FITR spectrometer (4700, Jasco) (Supplementary Figure S1). Cross sections were obtained from the HITRAN database.[8] The system was flushed for 30 minutes to remove air from the system prior to striking the plasma. A background spectrum was taken prior to the ignition of the plasma and all measurements were taken after the system had been in operation for 30 min and reached steady state (Supplementary Figure S2).

GCMS and NMR analysis: After plasma exposure, 500 µL samples of the treated solution were taken for gas chromatography mass spectrometry (GCMS-QP2010 Ultra/SE, Shimadzu) analysis. Samples were brought to room temperature and 50 µL of Naphthalene solution (50 mM) was added for quantitative analysis. The GCMS was equipped with a Rtx-5MS capillary column (30 m × 0.25 mm × 0.25 µm). A Shimadzu 40 Headspace Sampler was used to introduce the liquid samples automatically in the GCMS and this was

configured to use split injection and an injection volume of 1 μL. The carrier gas was He and the flow rate 65 mL/min (constant flow rate). The column was kept at 50°C for 1 min after injection, and the temperature was then increased to 180 °C at 20 °C/min, held at 180°C for 5 min and increased to 250 °C at 20 °C/min. The temperature was then held at 250°C for 2 min before cooling down, giving a total run time of 18 min per sample. Compounds labeled in the chromatographs have been identified not only by their mass spectra but also by comparing their retention time with purified commercially available samples and calibration curves matching the concentration of the most relevant compounds to the area observed in the chromatographs were obtained using purified commercial compounds (Supplementary Figure S8).

A.5. Computational details

Computations were performed at the multi-reference averaged quadratic coupled cluster (MR-AQCC) level of theory[9] using Dunning's cc-pVDZ basis set.[10] A complete active space (CAS) placing 6 electrons in 5 orbitals (ethene-π, ethene-π^*, 3 $\times$ oxygen-p) was chosen as a reference space. The orbitals were optimised using CASSCF employing the same active space. State-averaging was performed over 6 singlet and 3 triplet states in order to include all components of the ^1D and ^3P terms, respectively. Geometry optimisations at the MR-AQCC level[11] were performed individually on the S_1 and T_1 surfaces constraining the average CO distance and relaxing the remaining structure. Computations were performed using the program system Columbus.[12,13]

B Supplementary Figures

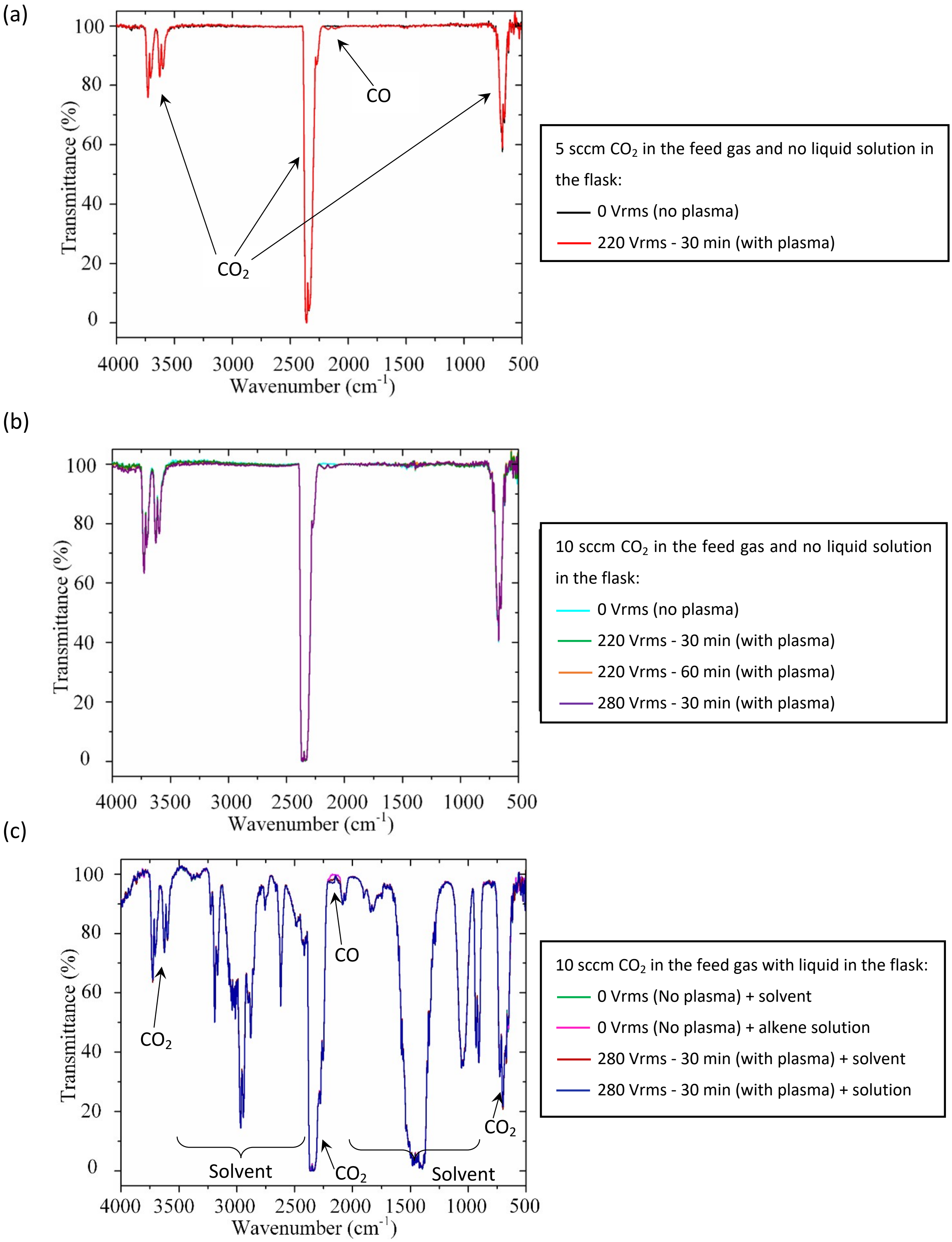

Figure S2. FTIR spectra of the gases at the exhaust for various experimental conditions. (a) Shows the formation of CO in the plasma in the absence of a liquid solution. (b) Shows that after 30 min the composition of the exhaust gas has reached steady state. (c) Shows that when liquids are introduced in the system, the IR spectra of the exhaust gases is dominated by solvent molecules. Crucially, however, no solvent peaks overlap with the CO absorption bands (2100–2250 cm^{-1}).

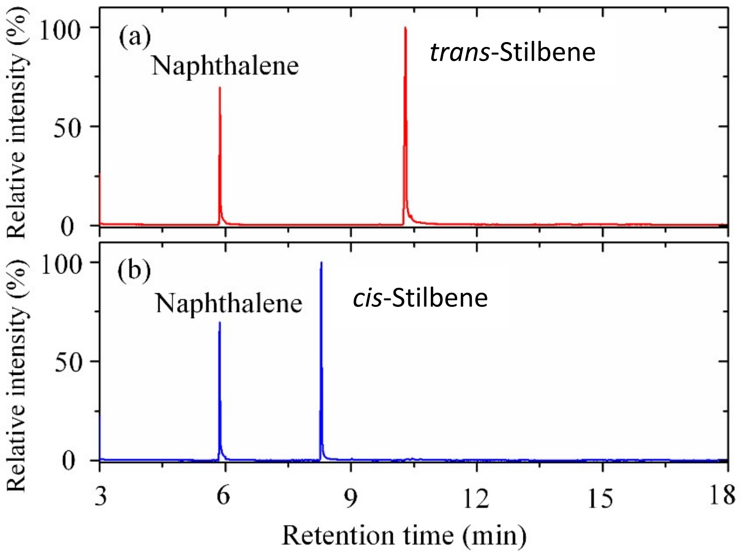

Figure S3. Chromatographs of *trans*-stilbene and *cis*-stilbene solution after 60 min exposure to a pure helium plasma. Voltage 250 Vrms, solution temperature –25°C.

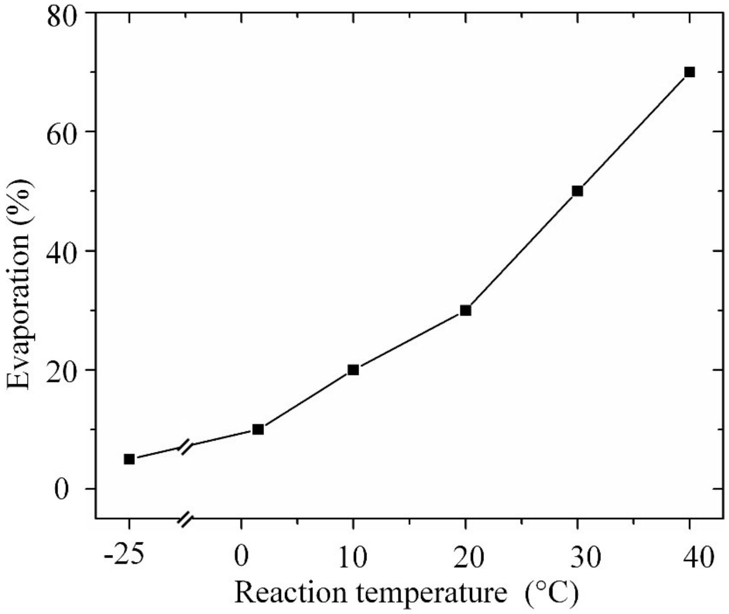

Figure S4. Solvent evaporation during a 20 min plasma treatment as a function of the liquid temperature. Plasma sustained at a voltage of 280 Vrms and with a CO_2 flow rate of 10 sccm.

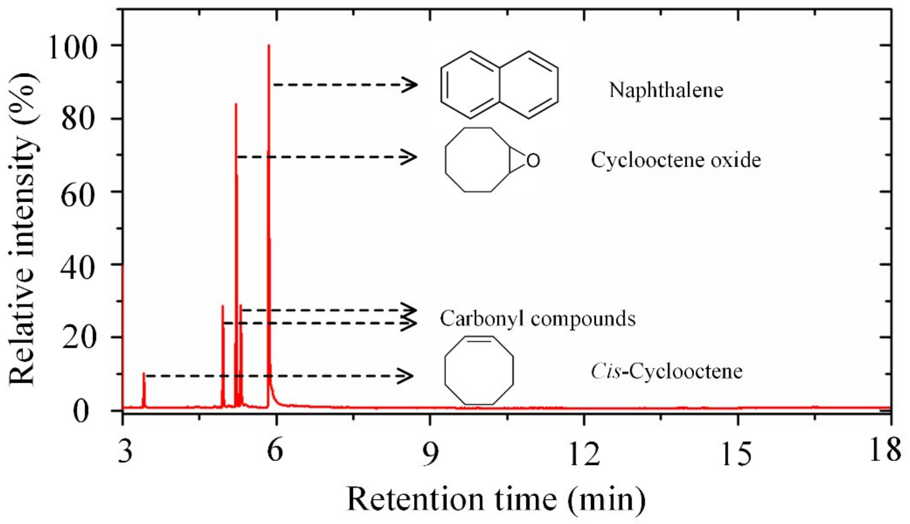

Figure S5. Chromatograph of the *cis*-cyclooctene solution after a 60 min exposure to He+CO_2 plasma. (280 Vrms, –25°C and 10 sccm CO_2)

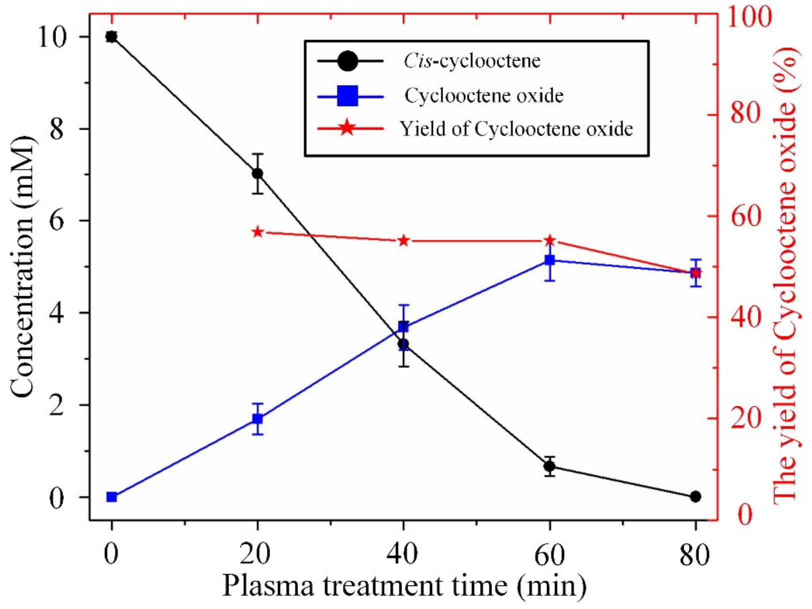

Figure S6. Time evolution of the concentration of *cis*-cyclooctene, cyclooctene oxide and the yield of cyclooctene oxide during exposure to He+CO_2 plasma (280 Vrms, –25°C and 10 sccm CO_2).

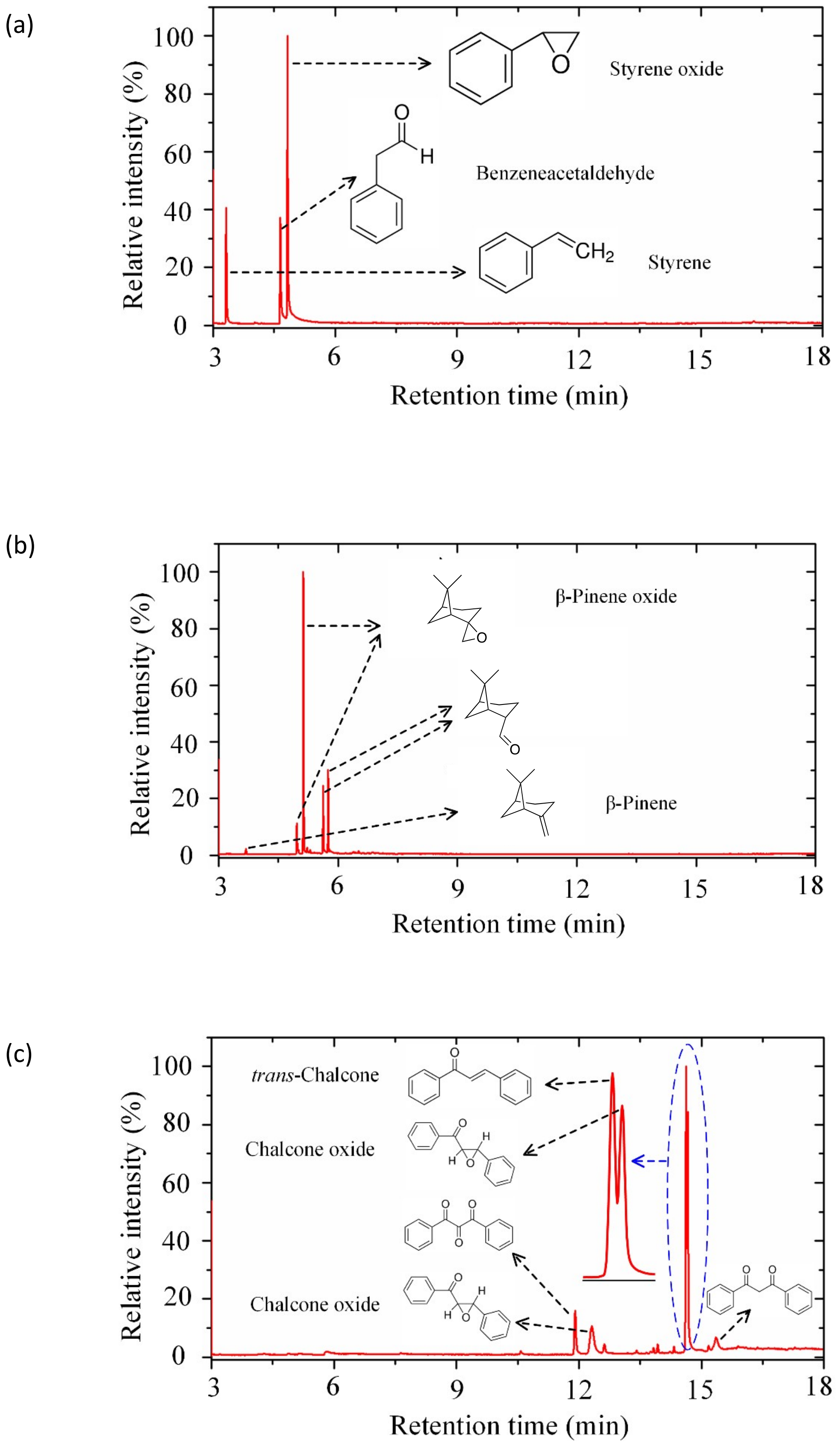

Figure S7. Chromatographs of (a) styrene, (b) β-pinene and (c) *trans*-chalcone solutions after a 60 min exposure to He+CO_2 plasma. (280 Vrms, –25°C and 10 sccm CO_2)

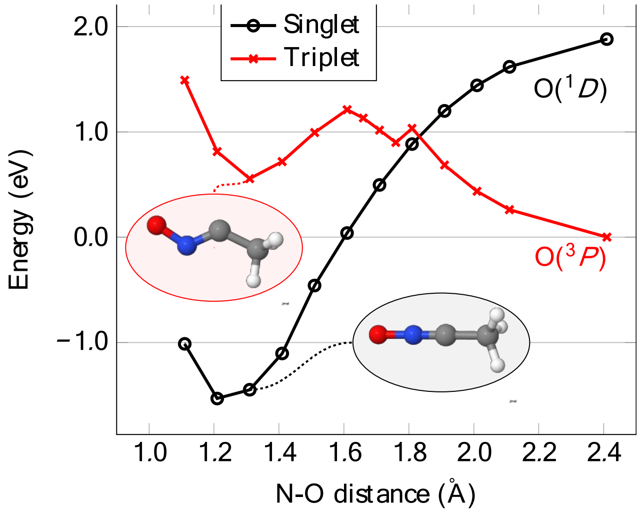

Figure S8. Potential energy curves for an oxygen atom approaching acetonitrile considering singlet (black) and triplet (red) spin computed at the ab initio MR-AQCC level of theory by fixing the N-O distance and relaxing the remaining structure. The minima on the singlet and triplet surfaces are shown as insets. O(^{1}D) readily reacts with acetonitrile, thereby quenching O(^{1}D). On the other hand, there is a 1.2eV barrier for O(^{3}P) to react with acetonitrile. This barrier prevents the spontaneous quenching of triplet oxygen by the solvent, making it available for epoxidation of the stilbene. These simulation results, together with the fact that we do not observe cycloaddition of nitrile oxides to the alkene support the idea that O(^{3}P) reacts directly with the alkene and not with the solvent.

(a)

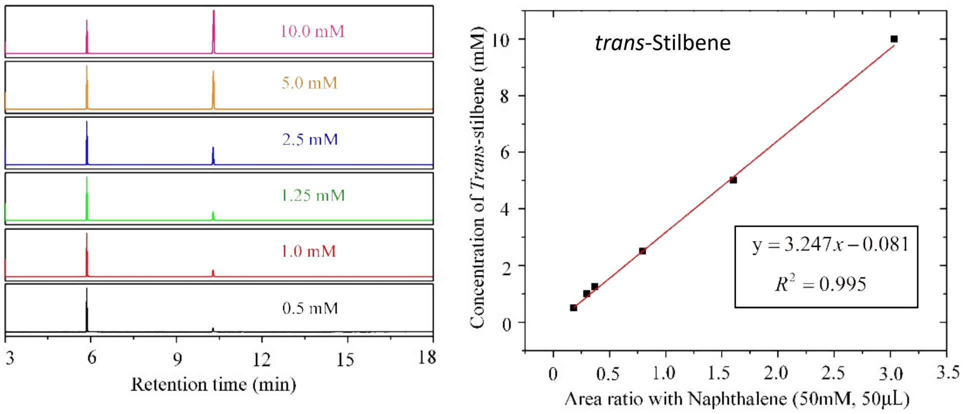

10.0 mM
5.0 mM
2.5 mM
1.25 mM
1.0 mM
0.5 mM
Retention time (min)
trans-Stilbene
$y = 3.247x - 0.081$
$R^2 = 0.995$
Concentration of *Trans*-stilbene (mM)
Area ratio with Naphthalene (50mM, 50μL)

(b)

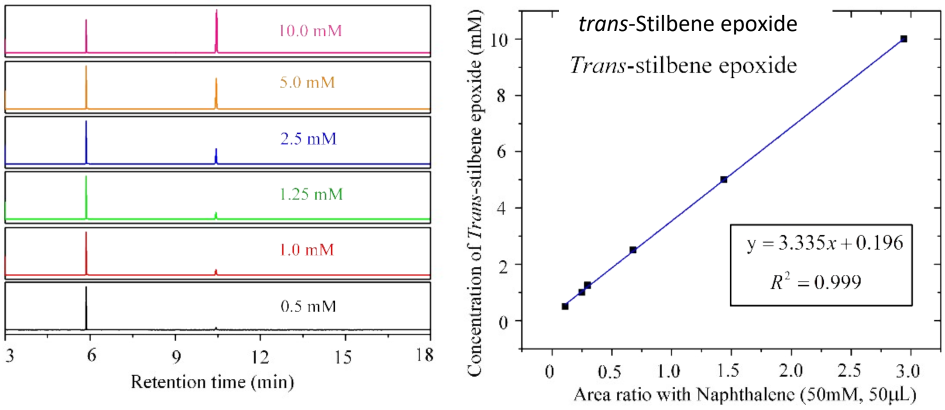

10.0 mM
5.0 mM
2.5 mM
1.25 mM
1.0 mM
0.5 mM
Retention time (min)
trans-Stilbene epoxide
Trans-stilbene epoxide
$y = 3.335x + 0.196$
$R^2 = 0.999$
Concentration of *Trans*-stilbene epoxide (mM)
Area ratio with Naphthalene (50mM, 50μL)

(c)

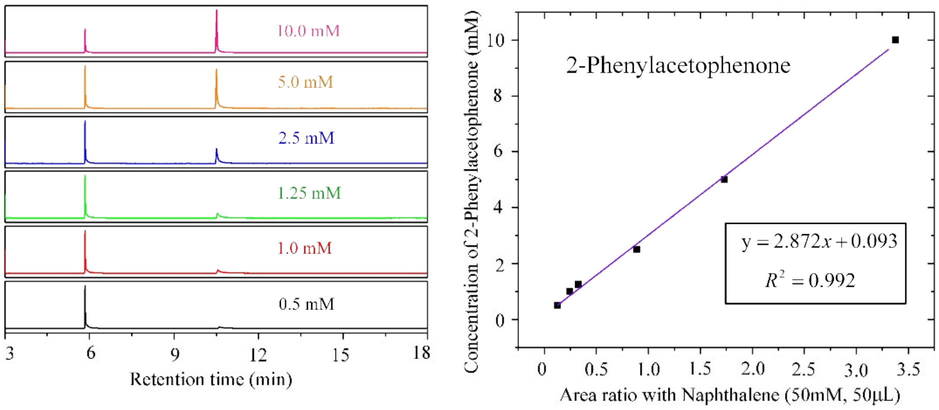

10.0 mM
5.0 mM
2.5 mM
1.25 mM
1.0 mM
0.5 mM
Retention time (min)
2-Phenylacetophenone
$y = 2.872x + 0.093$
$R^2 = 0.992$
Concentration of 2-Phenylacetophenone (mM)
Area ratio with Naphthalene (50mM, 50μL)

(d)

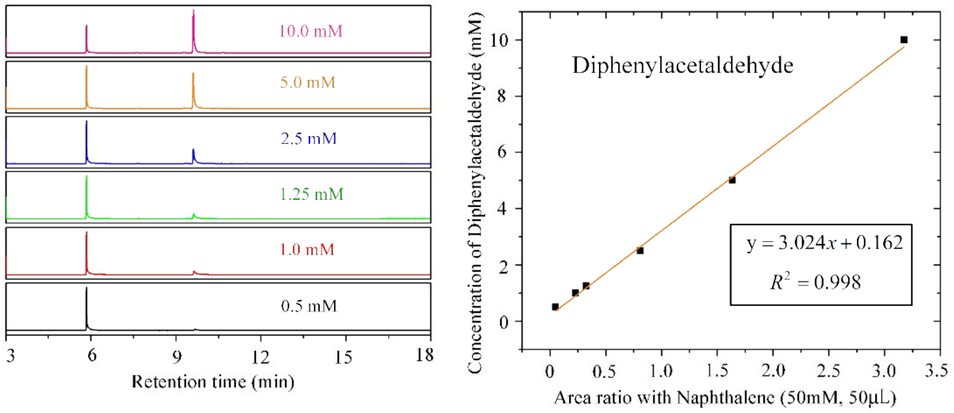

10.0 mM
5.0 mM
2.5 mM
1.25 mM
1.0 mM
0.5 mM
Retention time (min)
Diphenylacetaldehyde
$y = 3.024x + 0.162$
$R^2 = 0.998$
Concentration of Diphenylacetaldehyde (mM)
Area ratio with Naphthalene (50mM, 50μL)

(e)

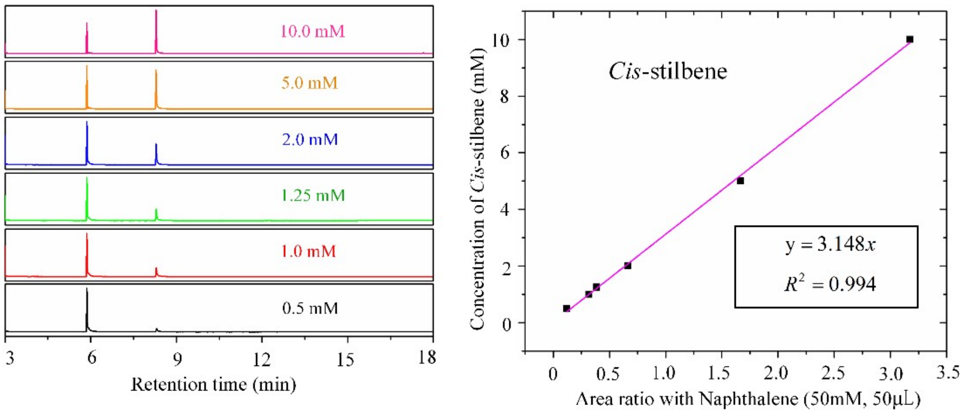
10.0 mM
5.0 mM
2.0 mM
1.25 mM
1.0 mM
0.5 mM
3 6 9 12 15 18
Retention time (min)
Cis-stilbene
y = 3.148x
R^2 = 0.994
Concentration of Cis-stilbene (mM)
Area ratio with Naphthalene (50mM, 50μL)

(f)

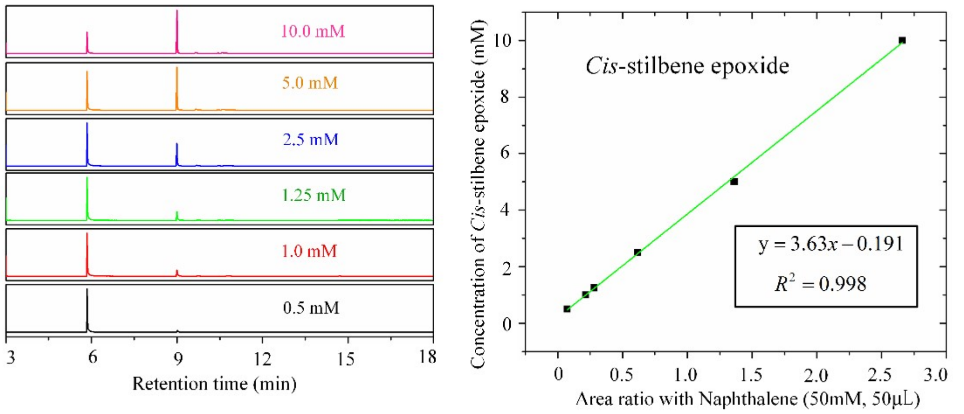
10.0 mM
5.0 mM
2.5 mM
1.25 mM
1.0 mM
0.5 mM
3 6 9 12 15 18
Retention time (min)
Cis-stilbene epoxide
y = 3.63x – 0.191
R^2 = 0.998
Concentration of Cis-stilbene epoxide (mM)
Area ratio with Naphthalene (50mM, 50μL)

(g)

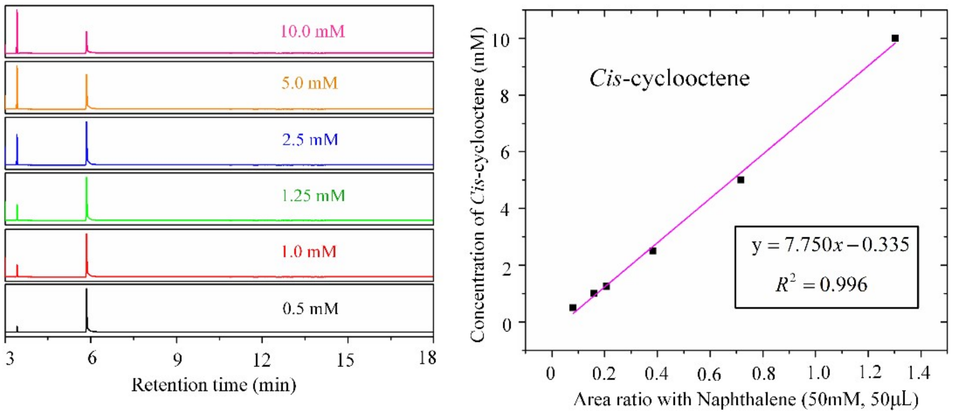
10.0 mM
5.0 mM
2.5 mM
1.25 mM
1.0 mM
0.5 mM
3 6 9 12 15 18
Retention time (min)
Cis-cyclooctene
y = 7.750x – 0.335
R^2 = 0.996
Concentration of Cis-cyclooctene (mM)
Area ratio with Naphthalene (50mM, 50μL)

(h)

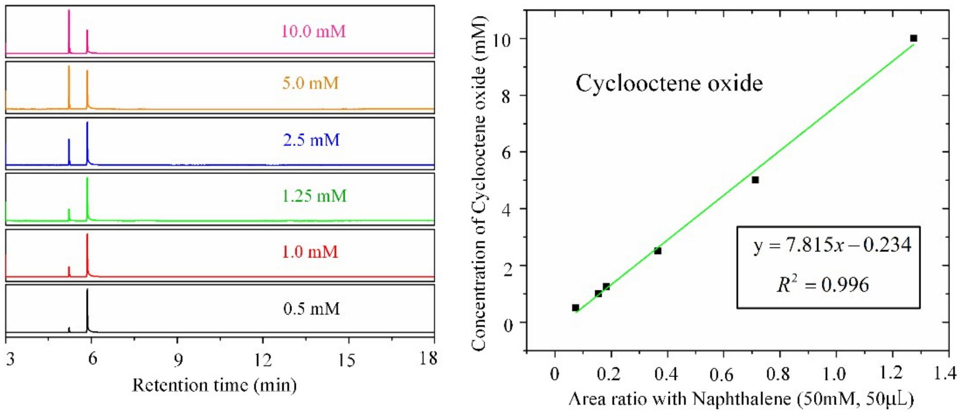
10.0 mM
5.0 mM
2.5 mM
1.25 mM
1.0 mM
0.5 mM
3 6 9 12 15 18
Retention time (min)
Cyclooctene oxide
y = 7.815x – 0.234
R^2 = 0.996
Concentration of Cyclooctene oxide (mM)
Area ratio with Naphthalene (50mM, 50μL)

Figure S9. Retention time and intensity calibration curves for (a) *trans*-stilbene, (b) *trans*-stilbene epoxide, (c) 2-Phenylacetophenone, (d) Diphenylacetaldehyde, (e) *cis*-stilbene, (f) *cis*-stilbene epoxide, (g) *cis*-cyclooctene and (h) cyclooctene oxide. All data obtained from solutions prepared with purified commercial samples (500 μL) and naphthalene (50 mM, 50 μL) as a reference.

C References

1. Golda, J. *et al.* Concepts and characteristics of the 'COST Reference Microplasma Jet'. *J. Phys. D. Appl. Phys.* **49**, 084003 (2016).
2. Schulz-Von Der Gathen, V. *et al.* Optical diagnostics of micro discharge jets. *Contrib. to Plasma Phys.* **47**, 510–519 (2007).
3. Knake, N., Reuter, S., Niemi, K., Schulz-Von Der Gathen, V. & Winter, J. Absolute atomic oxygen density distributions in the effluent of a microscale atmospheric pressure plasma jet. *J. Phys. D. Appl. Phys.* **41**, 194006 (2008).
4. Ellerweg, D., Benedikt, J., von Keudell, A., Knake, N. & Schulz-von der Gathen, V. Characterization of the effluent of a He/O2 microscale atmospheric pressure plasma jet by quantitative molecular beam mass spectrometry. *New J. Phys.* **12**, 013021 (2010).
5. Gorbanev, Golda, Gathen & Bogaerts. Applications of the COST Plasma Jet: More than a Reference Standard. *Plasma* **2**, 316–327 (2019).
6. Benedikt, J. *et al.* The fate of plasma-generated oxygen atoms in aqueous solutions: non-equilibrium atmospheric pressure plasmas as an efficient source of atomic O (aq). *Phys. Chem. Chem. Phys.* **20**, 12037–12042 (2018).
7. Xu, H. *et al.* trans-Stilbene epoxidation by He+O2 atmospheric pressure plasma: Epoxidation without oxidant waste stream. *Plasma Process. Polym.* e1900162 (2019) doi:10.1002/ppap.201900162.
8. Gordon, I. E. *et al.* The HITRAN2016 molecular spectroscopic database. *J. Quant. Spectrosc. Radiat. Transf.* **203**, 3–69 (2017).
9. Szalay, P. G. & Bartlett, R. J. Multi-reference averaged quadratic coupled-cluster method: a size-extensive modification of multi-reference CI. *Chem. Phys. Lett.* **214**, 481–488 (1993).
10. Dunning, T. H. Gaussian basis sets for use in correlated molecular calculations. I. The atoms boron through neon and hydrogen. *J. Chem. Phys.* **90**, 1007–1023 (1989).
11. Lischka, H. *et al.* High-level multireference methods in the quantum-chemistry program system COLUMBUS: Analytic MR-CISD and MR-AQCC gradients and MR-AQCC-LRT for excited states, GUGA spin–orbit CI and parallel CI density. *Phys. Chem. Chem. Phys.* **3**, 664–673 (2001).
12. Lischka, H. *et al.* Columbus-a program system for advanced multireference theory calculations. *Wiley Interdiscip. Rev. Comput. Mol. Sci.* **1**, 191–199 (2011).
13. Lischka, H. *et al.* The generality of the GUGA MRCI approach in COLUMBUS for treating complex quantum chemistry. *J. Chem. Phys.* **152**, 134110 (2020).
